# Laboratory evaluation of plant powders against the maize weevil, *Sitophilus zeamais* (Coleoptera: Curculionidae), in stored sorghum

**DOI:** 10.1371/journal.pone.0357778

**Published:** 2026-09-11

**Authors:** Adem Nega Yimer, Bewketu Takele

**Affiliations:** Debre Markos University, College of Natural and Computational Sciences, Debre Markos, Ethiopia; Central Food Technological Research Institute CSIR, INDIA

## Abstract

Sorghum, (*Sorghum bicolor* L. Moench) one of the oldest cultivated cereals and a major staple food for millions of people in many tropical and subtropical regions, is highly vulnerable to postharvest losses caused by storage pests such as the maize weevil, *Sitophilus zeamais* Motschulsk. Although the insecticidal effects of several botanical powders against *S. zeamais* have been previously reported, evidences specific to stored sorghum remains limited. The present study confirmed and comparatively evaluated the insecticidal efficacy of three plant-based powders: *Phytolacca dodecandra* L’Her, *Nicotiana tabacum* L., and *Millettia ferruginea* Hochst. against adult maize weevils under laboratory conditions. Treatments were applied with varying concentrations (0.4, 0.6, 0.8 and 1.0g/100g) of each botanical powder, while untreated and 5% malathion dust were used as controls. Adult mortality, progeny emergence, grain damage, and weight losses were recorded over a 15 day period. Results showed that *P. dodecandra* caused the highest adult mortality (96.6 ± 5.7), followed by *N. tabacum* (83.3 ± 0.0), and *M. ferruginea* (53.3 ± 3.8). All botanicals powder significantly reduced grain damage, and weight loss compared to the untreated control. These findings confirm the effectiveness of these botanicals in stored sorghum and highlight their potential relevance as locally available, eco-friendly alternatives to synthetic insecticides in Ethiopian postharvest pest management. Further research is needed under farm-level storage conditions to validate their practical applicability.

## Introduction

Sorghum, *Sorghum bicolor* is a major staple food crop grown in Ethiopia’s lowland and moisture stressed regions, with an annual production of approximately 4.1 million tons [1,2]. It is highly valued by smallholder farmers because of its adaptability to water limited areas and its multiple uses. Nutritionally, *S. bicolor* is a key staple crop supporting food security for over 100 million people in the eastern Horn of Africa [3–5].

In traditional grain storage systems, farmers commonly use botanical powders derived from plants such as neem (*Azadirachta indica*), eucalyptus (*Eucalyptus globulus*), tobacco (*Nicotiana tabacum*), garlic (*Allium sativum*), and hot pepper (Capsicum spp.) as eco-friendly protectants against stored insect pests [6–8].

Despite its importance, large amount of stored *S. bicolor* are lost annually to insect pests, with the maize weevil, *Sitophilus zeamais*, being the most destructive [5,9,10]. Infestation usually starts in the field, but the most significant damage occurs during storage. In Ethiopia, heavy infestation of stored *S. bicolor* can result in weight losses ranging from 41% to 48% under traditional storage systems [5]. Although synthetic insecticides are effective and convenient in minimizing storage loss, there is a global concern about environmental pollution and pesticide residues in food [10,11]. Moreover, their cost and limited accessibility reduce their suitability for low-income farmers, who may rely on cheaper but hazardous products. These challenges have stimulated growing interest in botanically derived pest control agents as safer and more sustainable alternatives for stored products protection. Numerous plant species contain biologically active secondary metabolites such as alkaloids, terpenoids, phenolics, and saponins that can affect insect survival, reproduction, and behavior.

Previous studies have demonstrated that botanical powders and extracts can effectively suppress insect pests in stored sorghum by causing adult mortality, reducing oviposition, inhibiting progeny emergence, and limiting grain damage [10,12–14]. However, comparative biological information on the efficacy of locally available plant powders against *S. zeamais*, particularly with respect to both adult and progeny level effects, remains limited under Ethiopian conditions. Moreover, the mechanisms through which different botanical powders exert their effect whether through direct toxicity, physical abrasion, repellence, or reproductive inhibition are often inferred rather than explicitly examined.

Several studies have previously demonstrated the insecticidal activity of botanical powders such as *Millettia ferruginea*, *Nicotiana tabacum*, and *Phytolacca dodecandra* against stored product pests, including *S. zeamais* [12–14]. However, most of these investigations have focused on maize, while information on their efficacy in stored sorghum remains limited. In addition, comparative evaluations of these botanicals under uniform experimental condition are relatively scarce. Therefore, the objective of the present study was to confirm and comparatively evaluate the insecticidal effects of selected plant powders against *S. zeamais* in stored sorghum under laboratory conditions, with particular emphasis on adult mortality, progeny emergence, grain damage, and weight loss. By providing crop-specific and regionally relevant data, this study aims to support evidence-based use of locally available botanicals in sustainable sorghum stored pest management.

## Materials and methods

The study was conducted from April to June 2023 at the Entomological Laboratory of the Kombolcha Plant Health Clinic (KPHC), located 375 km Northeast of Addis Ababa (11°6’ N latitude and 39°43’ E) at an elevation of 1492−2084 m.a.s.l. The area has an arid to semi arid climate, with an average temperature ranging from 13.2^0^C-26.3^0^C. The experiment was carried out under ambient laboratory conditions.

### Mass rearing of the test insects

Heavily infested *S. bicolor* grains (> 50% infestation) were collected from pesticide-free farmers’ stores in Kombolcha town. Grains were kept in the laboratory for 3–6 days to allow adult emergence. The weevils were screened and identified using standard morphological identification keys described by [15] and the species identity was confirmed as *S. zeamais* based on diagnostic taxonomic characteristics. Adults were reared on disinfected sorghum (frozen at −20 ± 2°C for 2 weeks) to obtain uniform aged insects [4,14]. Five pairs of unsexed adult *S*. *zeamais* were introduced into 2 L glass jars containing 500 gm of sorghum grains. The jars were covered with muslin cloth tied with rubber bands to prevent escape while allowing aeration and maintained at 27 ± 2^o^C and 60–70 RH. Adults were allowed to oviposit on disinfested grains for 7–10 days in the dark [2], and then removed by sieving, and infested grains incubated for 40 days for growth. Newly emerged adults were collected daily and transferred to fresh grains to maintain a uniform population of 3–6-day old adults for bioassays.

### Collection and preparation of botanicals

The leaves of *Nicotiana tabacum*, the seeds of *Millettia ferruginea* and *Phytolacca dodecandra* were collected from natural and non-cultivated habitats. The plant species were identified and taxonomically authenticated using standard floristic keys and comparisons with authenticated herbarium specimens by a plant taxonomist at national herbarium of Ethiopia [16]. The plant materials were washed with tap water to remove debris and shade-dried for two weeks [17,18]. After drying, they were crushed with a mortar and pestle, and then ground into fine powder using an electric blender (RRH-A200, Shanghai Yuanya Industries and Trade Co. Ltd., China) operating at 28000 rpm. The resulting powders were mixed with sorghum grains on a weight-by-weight (w/w) basis, at concentrations of 0.4, 0.6, 0.8 and 1.0 g/100g of grains. Cropsave malathion 5% dust used as the positive control was purchased from a local agricultural supplier in Addis Ababa, Ethiopia applied at a rate of 0.5 g/kg of grain (equivalent to 25 mg active ingredient per kg grain). An untreated control was included as a negative control.

### Permits and permissions

No specific permits were required for this study. Plant materials and insect infested sorghum grains were collected from non-protected areas and privately owned farmers’ stores with local consent. The study did not involve endangered or protected species, and therefore formal approval from a governmental or regulatory authority was not required.

### Disinfesting of sorghum seeds for the study

Local sorghum grains were obtained from a market and disinfected by freezing at −4^0^C for 7 days [19] to eliminate any live insects. The grains were then air-dried, washed, and manually graded, with only large and undamaged grains selected for the experiment.

### Treatments application and experimental designs

The experiment was conducted under controlled temperature and humidity, matching the weevil rearing conditions. Fourteen treatments were tested, including 3 botanicals, an untreated control and 5% malathion dust as a standard check. Each treatment was applied at 4 concentrations (0.4, 0.6, 0.8 and 1.0g per 100g of sorghum) in 1L jars, replicated 3 times in a completely randomized design (CRD) (S1 Table). The jars were shaken thoroughly for 5 minutes to ensure uniform coating, and then 10 freshly emerged adult weevils of the same age were introduced per jar [20]. Jars were placed 5 cm apart on a flat table and left undisturbed under ambient laboratory conditions (approximately 24–28°C natural photoperiod) for 20 days for oviposition. Observations were made on adult mortality, F1 progeny emergence, grain damage, and weight loss.

### Description of treatments and controls

Malathion 5% dust (0.5 g/kg of grain) was used as a positive control at its recommended laboratory application rate to provide a standard benchmark for comparisons. The concentration was not weight-equivalent to the botanical treatments (0.4–1.0 g/100 g grain), because plant powders are crude materials containing variable quantities of bioactive compounds. Therefore, comparisons were based on biological efficacy rather than equal formulation dosage, following standard practice in stored-product insect bioassay [21,22].

### Adult mortality bioassays

Adult mortality was recorded on days 1, 2, 3, 5, 10 and 15 after treatment, with dead insects counted and removed. Weevils were considered dead if unresponsive to gentle probing or if their legs and wings were not folded over the body. Cumulative mortality was calculated, and the percentage determined using the formula of Kabir *et al*. [23].


$$\begin{array}{c} \text{Mortality }(\%)=\frac{\text{Cumulative number of dead weevils}}{\text{Total number of weevils }}\times \text{1}\text{0}\text{0}\% \end{array}$$

The mortality rate was corrected and calculated using Abbott’s formula [24].


$$\begin{array}{c} 𝐂𝐨𝐫𝐫𝐞𝐜𝐭𝐞𝐝\;𝐦𝐨𝐫𝐭𝐚𝐥𝐢𝐭𝐲\;\left(\%\right)=\frac{\%\;𝐦𝐨𝐫𝐭𝐚𝐥𝐢𝐭𝐲\;𝐢𝐧\;𝐭𝐡𝐞\;𝐭𝐞𝐬𝐭-\%\;𝐦𝐨𝐫𝐭𝐚𝐥𝐢𝐭𝐲\;𝐢𝐧\;𝐭𝐡𝐞\;𝐜𝐨𝐧𝐭𝐫𝐨𝐥}{100-\%\;𝐦𝐨𝐫𝐭𝐚𝐥𝐢𝐭𝐲\;𝐢𝐧\;𝐜𝐨𝐧𝐭𝐫𝐨𝐥}\times 100\% \end{array}$$

### F1 progeny emergency test

Twenty days after adult introduction, all insects were removed, and grains were returned to their respective jars to further assess F_1_ progeny emergence. Counts were made on days 21, 22, 23, 25, 30 and 35 by sieving and inspecting the grains. Percentage reduction in adult emergence or inhibition rate (% IR) was calculated following Sabbour *et al*. [25].


$$\begin{array}{c} \%\text{ progeny emergence}=(\text{Cn}-\text{Tn})\text{ x 100}/\text{Cn} \end{array}$$

Where Cn is the number of newly emerged insects in the untreated jar and Tn is the number of insects in the treated jar.

### Damaged grains

Grain damage was assessed on the 45^th^ day after adult *S. zeamais* introduction. Ten grains were randomly sampled from each jar and examined for signs of damage such as insect emergence holes. The proportion of damaged grains was calculated to estimate the extent of grain damage.


$$\begin{array}{c} \text{Damaged Grain }(\%)=\frac{\text{Number of damaged grains}}{\text{Tota number of grains }}\times \text{1}\text{0}\text{0}\% \end{array}$$

### Grain weight loss

Percent weight loss was determined by the count and weighs method as recommended by Adams and Schlten [26] and calculated by using the formula:


$$\begin{array}{c} \text{Weight loss }(\%)=\;\frac{\left(\text{Wu }\times \text{Nd})-(\text{Wd}\times \text{Nu}\right)}{\text{Wu }\times \;(\text{Nd Nu})}\text{x100} \end{array}$$

Where; Wu = Weight of undamaged grain, Nu = Number of undamaged grain, Wd = Weight of damaged grain, Nd = Number of damaged grain.

### Data analysis

Data were analyzed using SPSS version 21.0. Percentage mortality was angularly transformed, and progeny counts, grain damage, and weight losses were square root transformed to stabilize variances. One-way ANOVA assessed treatment effects, and means were compared using Tukey’s HSD test at the 5% significance. Transformed data were examined for normality and homogeneity of variances prior to analysis.

## Results

### Time dependent and mean percentage mortality of *S. zeamais* adults

All treatments significantly (P < 0.05) increased adult mortality with rising concentration and exposure time. *P. dodecandra* caused the highest mortality (96.6 ± 5.7%) after 15 days, followed by *N. tabacum* (83.3 ± 0.0%). *M. ferruginea* showed lower efficacy but still caused notable mortality at higher concentration (S1 Fig and Table 1).

**Table 1:** Mean percentage mortality (±SE) of adult *Sitophilus zeamais* exposed to *Phytolacca dodecandra, Nicotiana tabacum* and *Millettia ferruginea* powders (0.4-1.0 g/100 g grain), compared with malathion 5% dust (0.05 g/100 g grain) as a positive control and untreated grains as a negative controls.

Treatments	Concentrations (g/100g grain)	Mean % mortality ±SE at:					
		Day 1	Day 2	Day 3	Day 5	Day 10	Day 15
*M. ferruginea*	0.4	0.0 ± 0.0^a^	7.0 ± 1.2^ab^	12.0 ± 1.8^b^	17.0 ± 2.0^c^	22.0 ± 2.5^d^	35.0 ± 3.0^e^
	0.6	0.0 ± 0.0^a^	6.0 ± 1.0^ab^	11.0 ± 1.6^b^	16.0 ± 1.9^c^	21.0 ± 2.2^d^	33.0 ± 2.8^e^
	0.8	4.0 ± 0.8^a^	12.0 ± 1.5^b^	1.9 ± 1.9^c^	22.0 ± 2.3^d^	27.0 ± 2.6^e^	48.0 ± 3.5^f^
	1.0	5.0 ± 0.9^a^	11.0 ± 1.3^b^	17.0 ± 1.8^c^	23.0 ± 2.0^d^	29.0 ± 2.7^e^	53.3 ± 3.8^f^
*N. tabacum*	0.4	0.0 ± 0.0^a^	9.0 ± 0.0^a^	11.3 ± 3.3^b^	20.0 ± 5.7^bc^	30.3 ± 3.3^c^	41.0 ± 5.7^d^
	0.6	0.0 ± 0.0^a^	11.0 ± 0.0^a^	17.0 ± 0.0^a^	23.0 ± 5.7^bc^	34.6 ± 3.3^cd^	44.6 ± 3.3^cd^
	0.8	0.0 ± 0.0^a^	13.0 ± 0.0^a^	15.0 ± 0.0^a^	30.6 ± 3.3^ab^	46.6 ± 3.3^ab^	63.3 ± 3.3^c^
	1.0	0.0 ± 0.0^a^	15.0 ± 0.0^a^	21.0 ± 0.0^a^	37.0 ± 0.0^a^	57.0 ± 0.0^a^	83.3 ± 0.0^a^
*P. dodecandra*	0.4	0.0 ± 0.0^a^	12.0 ± 2.0^a^	21.0 ± 2.6^b^	32.0 ± 3.1^c^	45.00 ± 3.8^bc^	40.0 ± 4.2^c^
	0.6	0.0 ± 0.0^a^	16.0 ± 2.3^a^	25.0 ± 2.7^a^	36.0 ± 3.3^ab^	46.0 ± 3.6^d^	55.0 ± 4.5^ab^
	0.8	0.0 ± 0.0^a^	18.0 ± 2.5^a^	28.0 ± 2.9^a^	35.0 ± 3.2^ab^	57.0 ± 3.9^a^	78.0 ± 5.3^a^
	1.0	9.0 ± 1.8^a^	20.0 ± 2.6^a^	28.0 ± 2.8^a^	43.0 ± 3.6^a^	70.0 ± 4.8^a^	96.6 ± 5.7^a^
Malathion 5%	0.05	100.0 ± 2.8^a^	100.0 ± 2.8^a^	100.0 ± 2.8^a^	100.0 ± 2.8^a^	100.0 ± 2.8^a^	100.0 ± 2.8^a^
Untreated control		0.0 ± 0.0^a^	0.0 ± 0.0^a^	0.0 ± 0.0^a^	0.0 ± 0.0^a^	0.0 ± 0.0^a^	0.0 ± 0.0^a^

Means within the columns followed by the same letters are not significantly different (ANOVA with Tukey’s HSD, P < 0.05). Zero standard error values indicate identical observations among replicates at specific treatment levels.

### Cumulative mortality of adult weevils exposed to *P. dodecandra* powder

Cumulative mortality increased progressively with both concentration and exposure time. Mortality was initially low but rose sharply after several days, reaching nearly 100% at 1.0g/100 g grain after 15 days comparable to the synthetic control. This trend confirms a strong dose- and time-dependent toxic effect of the *P. dodecandra* powder (S2 Fig).

### Effect of botanical powders on F1 progeny emergence of *S. zeamais*

All botanical powders significantly (P < 0.05) reduced F1 progeny emergence compared to the untreated control. The untreated control had the highest (36.6.0 ± 5.7) emergence on day 35. In contrast, *P. dodecandra,* and *N. tabacum* completely inhibited progeny emergence at 1.0g/100g, matching the efficacy of 5% malathion dust. *M. ferruginea* showed moderate suppression, mainly at higher concentrations (Table 2).

**Table 2:** Mean number of F1 progeny (±SE) of *Sitophilus zeamais* emerged from sorghum treated with *Phytolacca dodecandra, Nicotiana tabacum* and *Millettia ferruginea* powders (0.4-1.0 g/100 g grain), with malathion 5% dust (0.05 g/100 g grain) and untreated grains as controls.

Treatments	Concentrations (g/100g grain)	Mean number of F1 progeny ±SE					
		Day 21	Day 22	Day 23	Day 25	Day 30	Day 35
*M. ferruginea*	0.4	0.0 ± 0.0^a^	0.0 ± 0.0^a^	6.6 ± 3.3^ab^	16.6 ± 3.3^b^	10.0 ± 5.7^abc^	23.3 ± 6.6^b^
	0.6	0.0 ± 0.0^a^	0.0 ± 0.0^a^	3.3 ± 3.3cd	20.0 ± 5.78^b^	3.3 ± 3.3cd	13.3 ± 3.3^ef^
	0.8	0.0 ± 0.0^a^	0.0 ± 0.0^a^	0.0 ± 0.0^a^	13.3 ± 3.3^ef^	6.6 ± 6.6^ab^	6.6 ± 6.6^ab^
	1.0	0.0 ± 0.0^a^	0.0 ± 0.0^a^	0.0 ± 0.0^a^	0.0 ± 0.0^a^	0.0 ± 0.0^a^	3.3 ± 3.3cd
*N. tabacum*	0.4	0.0 ± 0.0^a^	0.0 ± 0.0^a^	3.3 ± 3.3^cd^	10.0 ± 5.7^abc^	13.3 ± 3.3^ef^	20.0 ± 5.7^b^
	0.6	0.0 ± 0.0^a^	0.0 ± 0.0^a^	0.0 ± 0.0^a^	10.0 ± 5.7^abc^	16.6 ± 3.3^b^	16.6 ± 3.3^b^
	0.8	0.0 ± 0.0^a^	0.0 ± 0.0^a^	0.0 ± 0.0^a^	6.6 ± 3.3^ab^	6.6 ± 3.3^ab^	13.3 ± 3.3^ef^
	1.0	0.0 ± 0.0^a^	0.0 ± 0.0^a^	0.0 ± 0.0^a^	0.0 ± 0.0^a^	0.0 ± 0.0^a^	0.0 ± 0.0^a^
*P. dodecandra*	0.4	0.0 ± 0.0^a^	0.0 ± 0.0^a^	3.3 ± 3.3^cd^	16.6 ± 3.3^b^	10.0 ± 5.7^abc^	13.3 ± 3.3^ef^
	0.6	0.0 ± 0.0^a^	0.0 ± 0.0^a^	0.0 ± 0.0^a^	6.6 ± 3.3^ab^	20.0 ± 5.7^b^	3.3 ± 3.3^cd^
	0.8	0.0 ± 0.0^a^	0.0 ± 0.0^a^	0.0 ± 0.0^a^	3.3 ± 3.3cd	0.0 ± 0.0^a^	0.0 ± 0.0^a^
	1.0	0.0 ± 0.0^a^	0.0 ± 0.0^a^	0.0 ± 0.0^a^	0.0 ± 0.0^a^	0.0 ± 0.0^a^	0.0 ± 0.0^a^
Malathion 5%	0.05	0.0 ± 0.0^a^	0.0 ± 0.0^a^	0.0 ± 0.0^a^	0.0 ± 0.0^a^	0.0 ± 0.0^a^	0.0 ± 0.0^a^
Untreated control	–	0.0 ± 0.0^a^	0.0 ± 0.0^a^	10.0 ± 5.7^ac^	13.3 ± 3.3^ef^	16.6 ± 3.3^b^	36.6 ± 5.7^ac^

Within each column, means followed by the same superscript letter are not significantly different (ANOVA with Tukey’s HSD, P < 0.05).

### Effect of botanical powders on sorghum grain damage

All botanical powders significantly (p < 0.05) reduced grain damage compared to the untreated control. *Phytolacca dodecandra* completely prevented damage (at all concentrations) as did the malathion 5% dust. *Nicotiana tabacum* and *M. ferruginea* showed dose-dependent protection, with minimal or no damage at higher concentrations (Table 3).

**Table 3:** Mean percentage grain damage (± SE) of sorghum caused by *Sitophilus zeamais* after *Phytolacca dodecandra, Nicotiana tabacum* and *Millettia ferruginea* powders (0.4-1.0 g/100 g grain), with malathion 5% dust (0.05 g/100 g grain) and untreated grains as controls.

	Concentration (g/100g grain)	Mean % damage ± SE		
		*M. ferruginea*	*N. tabacum*	*P. dodecandra*
	0.4 g	1.00 ± 0.5^b^	0.67 ± 0.6^b^	0.00 ± 0.0^b^
	0.6 g	0.67 ± 3.3^b^	0.33 ± 0.3^c^	0.00 ± 0.0^b^
	0.8 g	0.33 ± 3.3^c^	0.0 ± 0.0^c^	0.00 ± 0.0^b^
	1.0 g	0.00 ± 0.0^c^	0.0 ± 0.0^c^	0.00 ± 0.0^b^
Malathion 5%	0.05g	0.00 ± 0.0^c^	0.00 ± 0.0^c^	0.00 ± 0.0^b^
Untreated control	–	2.00. ± 0.5^a^	3.0 ± 1.1^a^	2.67 ± 0. 3^a^

Within each column, means followed by the same superscript letter are not significantly different (ANOVA with Tukey’s HSD, P< 0.05).

### Effect of botanical powders on sorghum grain weight loss

All treatments significantly (p < 0.05) reduced sorghum grain weight loss compared to the untreated control, with protection improving at higher concentrations. *P. dodecandra* completely prevented weight loss at all levels, while *N. tabacum* showed strong, dose-dependent reduction. The untreated controls recorded the highest loss (3.67 ± 1.2%) indicating sever weevil damage (Table 4).

**Table 4:** Mean percentage weight loss (± SE) of sorghum grains caused by *Sitophilus zeamais* following *Phytolacca dodecandra, Nicotiana tabacum* and *Millettia ferruginea* powders (0.4-1.0 g/100 g grain), with malathion 5% dust (0.05 g/100 g grain) and untreated grains as controls.

	Concentrations (g/100g of seeds)	Mean % weight loss ± SE		
		*M. ferruginea*	*N. tabacum*	*P. dodecandra*
	0.4 g	1.67 ± 0.3^b^	1.00 ± 0.58^b^	0.00 ± 0.0^b^
	0.6 g	1.00 ± 0.58^b^	0.33 ± 0.3^c^	0.00 ± 0.0^b^
	0.8 g	0.67 ± 0.3^b^	0.33 ± 0.3^c^	0.00 ± 0.0^b^
	1.0 g	0.33 ± 0.3^c^	0.00 ± 0.0^c^	0.00 ± 0.0^b^
Malathion 5%	0.05 g	0.00 ± 0.0c	0.00 ± 0.0c	0.00 ± 0.0b
Untreated control	–	2.00 ± 0.58^a^	3.67 ± 1.20^a^	3.00 ± 0.58^a^

Within each column, means followed by the same superscript letter are not significantly different (ANOVA with Tukey’s HSD, P< 0.05).

## Discussion

The present study largely confirms the strong insecticidal activity of *P. dodecandra* powder against *S. zeamais*, with efficacy increasing in response to both concentration and exposure duration. At the highest concentration (1.0g/100g of grain) *P. dodecandra* caused nearly complete adult mortality within 15 days, achieving an efficacy comparable to 5% malathion dust. This clear dose-and-time dependent response indicates that increased quantities of powder enhance insect exposure and cumulative toxic effects during storage. Similar concentration-dependent effects have been widely reported for botanical powders used against stored product pests, in accordance to the present findings [27,28].

Adult mortality increased progressively overtime across all botanical treatments, suggesting a residual mode of action rather than an acute knockdown effect. Such delayed toxicity is commonly observed with plant based powders applied to stored grains and is often attributed to prolonged contact with treated kernels and continuous physiological stress on insects [25,27,28]. Although the present study did not investigate the specific mode of action directly, the observed time-dependent lethality is consistent with earlier reports on the sustained effectiveness of botanical powders on storage system.

*Nicotiana tabacum* powder also showed strong insecticidal activity, showing a clear dose- and time-dependent mortality patterns, though its efficacy was lower than that of *P. dodecandra*. These results align with previous studies reporting significant mortality of stored grain weevils following treatment with *N. tabacum* powder at higher application rates [10,29]. In contrast, *M. ferruginea* showed comparatively lower adult mortality but still caused significant effects at higher concentrations, confirming a measurable dose or concentrations response relationship. Similar trends have been reported in earlier studies where higher dosage of *M. ferruginea*-based materials resulted in increased mortality of storage insect pests [28,30].

Progeny emergency was strongly suppressed by botanical powder treatments, particularly at higher concentrations of *P. dodecandra* and *N. tabacum*, where no F1 adults emerged. In contrast the untreated control and grains treated with the lowest concentration of *M. ferruginea* recorded the highest progeny counts by day 35. These results clearly indicate that botanical powders effectively disrupt population development of *S. zeamais* during storage. Although the present study did not examine specific developmental stages, concentration-dependent reduction in progeny emergence suggests interference with reproduction and immature development. Comparable suppression of progeny emergence by plant-derived powders has been widely documented for stored-product insects [29,31].

All botanical powders significantly reduced sorghum grain damage and weight loss compared to the untreated control, with protective effects improving as concentrations increased. *Phytolacca dodecandra* provided complete protection against grain damage and weight loss at all tested rates, reflecting its strong overall performance in adult mortality and progeny suppression. *Nicotiana tabacum and M. ferruginea* also minimized grain damage and weight loss in a dose-dependent manner, indicating enhanced protection at higher application rates. These results agree with previous reports by showing that increasing dosages of botanical powders improve grain protection and reduce post-harvest losses caused by weevil infestation [32].

The findings of the present study agree with earlier reports demonstrating the insecticidal potential of botanical powders against *S. zeamais.* In the present study, *P. dodecandra* and *N. tabacum* powders caused 96.6% and 83.3% adult mortality, respectively at 1.0 g/100 g of grain after 15 days of exposure. Comparable results were reported by Ogendo *et al*. [33], were powders of *Tephrosia vogelii* and *Lantana camara* caused 85.0–93.7% and 82.7–90.0% mortality, respectively, against *S. zeamais* after 21 days of exposure. Silva *et al*. [34] also reported 97.1–98.8% mortality using *Peumus boldus* powder at 1–2% (w/w). Similarly, studies conducted in Ethiopia indicated that *Azadirachta indica* powder caused up to 99% mortality *sitophilus* species at higher application rates [35]. The high efficacy observed in the present study may be associated with bioactive phytochemicals such as alkaloids, saponine, rotenoids, and nicotine compounds present in the tested plant species, which may exert toxic, repellent, and feeding deterrent effects on stored-product insects [36,37].

Overall the results of this study confirm that botanical powders, particularly *P. dodecandra,* are effective in reducing adult survival, suppressing progeny emergence, and minimizing grain damage and weight loss caused by *S. zeamais*. These attributes highlight their potential as sustainable alternatives to synthetic insecticide for stored grain protection in smallholder farming systems. However, further research is required to evaluate formulation stability, persistence under farmer storage conditions, and economic feasibility at the farm level. In addition, phytochemical characterization would strengthen mechanistic understanding. It is also important to note that these botanicals contain bioactive compounds that may pose risks to users and consumers depending on exposure and residue levels. Therefore, their safety should not be assumed, and further studies on toxicity, residue, dynamics and safe application practices are necessary despite the clear biological efficacy demonstrated in this study.

### Limitations of the study

Although this study provides useful comparative data on the efficacy of botanical powders against *S. zeamais* in stored sorghum, some limitations should be acknowledged. i) no inert powder or handling-only control was included, limiting separation of chemical and physical effects. ii) a small number of adults per replicate and lack of sexing may have influenced mortality and progeny outcomes, iii) One-way ANOVA was used for time dependent data, which may not fully capture interaction effects. Despite these limitations, replications and statistical analysis support the reliability of the comparative results and their relevance to sorghum storage protection.

## Conclusion

The present study confirms the effectiveness of *P. dodecandra* powder against *S. zeamais* in stored sorghum, as evidenced by high adult mortality, suppression of progeny emergence, and significant reductions in grain damage and weight loss. *N. tabacum* and *M. ferruginea* provided moderate levels of protection. Although malathion caused more rapid mortality, *P. dodecandra* achieved high mortality over longer exposure periods. These findings suggest the potential relevance of botanical powders particularly, *P. dodecandra,* for stored-grain pest management, though further study on formulation stability, long-term persistence, safety to humans and non-target organisms and on-farm applicability is needed. The observed effects may involve chemical or physical modes of action, which were not directly examined in this study.

## Supporting information

S1 Fig Mortality of *Sitophilus zeamais* adults exposed to botanical powders of *Phytolacca dodecandra, Nicotiana tabacum* and *Millettia ferruginea* with exposure times. (DOCX)

S2 Fig Cumulative mortality (%) trends of the insecticidal activity of *Phytolacca dodecandra* powder. (DOCX)

S1 Table Raw replicate level mortality data of *S. zeamais* (10 insects x 3 replicates) exposed to botanical powders at different exposure times. (DOCX)
