# Supplementary material for: Laboratory evaluation of plant powders against the maize weevil, Sitophilus zeamais (Coleoptera: Curculionidae), in stored sorghum
Source: PLoS One. 2026 Sep 11;21(9):e0357778. doi: 10.1371/journal.pone.0357778 (PMC13568508; doi:10.1371/journal.pone.0357778)
Supplement: Supplementary file 1 [file pone.0357778.s001.docx]

**SI File 1**

Botanical plant extracts

Mean % Weevils mortality

| Exposure time (days) | M. ferruginea | N. tabacum | P. dodecandra |
| --- | --- | --- | --- |
| 1 | 2.25 | 0 | 2.25 |
| 2 | 9 | 12 | 16.5 |
| 3 | 14.75 | 16 | 25.5 |
| 5 | 19.5 | 27.5 | 36.5 |
| 10 | 24.75 | 41.75 | 54.5 |
| 15 | 42.33 | 57.83 | 67.9 |

S1 Fig. **Mortality of** *Sitophilus* *zeamais* adults exposed to botanical powders of *Phytolacca dodecandra, Nicotiana tabacum* and *Millettia ferruginea* **with exposure times**
