# Supplementary material for: Laboratory evaluation of plant powders against the maize weevil, Sitophilus zeamais (Coleoptera: Curculionidae), in stored sorghum
Source: PLoS One. 2026 Sep 11;21(9):e0357778. doi: 10.1371/journal.pone.0357778 (PMC13568508; doi:10.1371/journal.pone.0357778)
Supplement: Supplementary file 2 [file pone.0357778.s002.docx]

**SI File 2**

Daily mean Weevils mortality (%±SE)

Weevils mortality at each rate (%)

| Exposure time (days) | 0.4 | 0.6 | 0.8 | 1 | Daily cumulative mean mortality | 'Commulative mean mortality (%)' | Mean cumulative mortality |
| --- | --- | --- | --- | --- | --- | --- | --- |
| 1 | 0 | 0 | 0 | 9 | 1 | 2.25 | 1 |
| 2 | 12 | 16 | 18 | 20 |  | 16.5 |  |
| 3 | 21 | 25 | 28 | 28 |  | 25.5 |  |
| 5 | 32 | 36 | 35 | 43 |  | 36.5 |  |
| 10 | 45 | 46 | 57 | 70 |  | 54.25 |  |
| 15 | 40 | 55 | 78 | 96.6 |  | 67.4 |  |

S2 Fig. **Cumulative mortality (%) trends of the insecticidal activity of** *Phytolacca dodecandra* **powder**
