# Supplementary material for: Laboratory evaluation of plant powders against the maize weevil, Sitophilus zeamais (Coleoptera: Curculionidae), in stored sorghum
Source: PLoS One. 2026 Sep 11;21(9):e0357778. doi: 10.1371/journal.pone.0357778 (PMC13568508; doi:10.1371/journal.pone.0357778)
Supplement: Supplementary file 3 [file pone.0357778.s003.docx]

**SI File 3**

S1 Table. Raw replicate level mortality data of *Sitophilus zeamais* (10 insects x 3
 replicates) exposed to botanical powders at different exposure times.

| No. | Treatments | Rates  (g/100g grain) | Replicates | Exposure times(days) | Adults exposed | Number dead |
| --- | --- | --- | --- | --- | --- | --- |
| 1 | *M. ferruginea* | 0.4 | 1 | 1 | 10 | 0 |
|  |  |  | 2 | 1 | 10 | 0 |
|  |  |  | 3 | 1 | 10 | 0 |
| 2 | *M. ferruginea* | 0.6 | 1 | 1 | 10 | 0 |
|  |  |  | 2 | 1 | 10 | 0 |
|  |  |  | 3 | 1 | 10 | 0 |
| 3 | *M. ferruginea* | 0.8 | 1 | 1 | 10 | 1 |
|  |  |  | 2 | 1 | 10 | 0 |
|  |  |  | 3 | 1 | 10 | 0 |
| 4 | *M. ferruginea* | 1 | 1 | 1 | 10 | 1 |
|  |  |  | 2 | 1 | 10 | 0 |
|  |  |  | 3 | 1 | 10 | 1 |
| 5 | *M. ferruginea* | 0.4 | 1 | 2 | 10 | 2 |
|  |  |  | 2 | 2 | 10 | 0 |
|  |  |  | 3 | 2 | 10 | 0 |
| 6 | *M. ferruginea* | 0.6 | 1 | 2 | 10 | 1 |
|  |  |  | 2 | 2 | 10 | 1 |
|  |  |  | 3 | 2 | 10 | 0 |
| 7 | *M. ferruginea* | 0.8 | 1 | 2 | 10 | 2 |
|  |  |  | 2 | 2 | 10 | 1 |
|  |  |  | 3 | 2 | 10 | 1 |
| 8 | *M. ferruginea* | 1 | 1 | 2 | 10 | 0 |
|  |  |  | 2 | 2 | 10 | 2 |
|  |  |  | 3 | 2 | 10 | 1 |
| 9 | *M. ferruginea* | 0.4 | 1 | 3 | 10 | 3 |
|  |  |  | 2 | 3 | 10 | 1 |
|  |  |  | 3 | 3 | 10 | 0 |
| 10 | *M. ferruginea* | 0.6 | 1 | 3 | 10 | 0 |
|  |  |  | 2 | 3 | 10 | 2 |
|  |  |  | 3 | 3 | 10 | 1 |
| 11 | *M. ferruginea* | 0.8 | 1 | 3 | 10 | 2 |
|  |  |  | 2 | 3 | 10 | 1 |
|  |  |  | 3 | 3 | 10 | 3 |
| 12 | *M. ferruginea* | 1 | 1 | 3 | 10 | 2 |
|  |  |  | 2 | 3 | 10 | 1 |
|  |  |  | 3 | 3 | 10 | 2 |
| 13 | *M. ferruginea* | 0.4 | 1 | 5 | 10 | 2 |
|  |  |  | 2 | 5 | 10 | 2 |
|  |  |  | 3 | 5 | 10 | 1 |
| 14 | *M. ferruginea* | 0.6 | 1 | 5 | 10 | 2 |
|  |  |  | 2 | 5 | 10 | 2 |
|  |  |  | 3 | 5 | 10 | 1 |
| 15 | *M. ferruginea* | 0.8 | 1 | 5 | 10 | 2 |
|  |  |  | 2 | 5 | 10 | 2 |
|  |  |  | 3 | 5 | 10 | 3 |
| 16 | *M. ferruginea* | 1 | 1 | 5 | 10 | 1 |
|  |  |  | 2 | 5 | 10 | 2 |
|  |  |  | 3 | 5 | 10 | 4 |
| 17 | *M. ferruginea* | 0.4 | 1 | 10 | 10 | 2 |
|  |  |  | 2 | 10 | 10 | 3 |
|  |  |  | 3 | 10 | 10 | 2 |
| 18 | *M. ferruginea* | 0.6 | 1 | 10 | 10 | 2 |
|  |  |  | 2 | 10 | 10 | 3 |
|  |  |  | 3 | 10 | 10 | 1 |
| 19 | *M. ferruginea* | 0.8 | 1 | 10 | 10 | 3 |
|  |  |  | 2 | 10 | 10 | 2 |
|  |  |  | 3 | 10 | 10 | 3 |
| 20 | *M. ferruginea* | 1 | 1 | 10 | 10 | 3 |
|  |  |  | 2 | 10 | 10 | 4 |
|  |  |  | 3 | 10 | 10 | 2 |
| 21 | *M. ferruginea* | 0.4 | 1 | 15 | 10 | 3 |
|  |  |  | 2 | 15 | 10 | 4 |
|  |  |  | 3 | 15 | 10 | 4 |
| 22 | *M. ferruginea* | 0.6 | 1 | 15 | 10 | 3 |
|  |  |  | 2 | 15 | 10 | 4 |
|  |  |  | 3 | 15 | 10 | 3 |
| 23 | *M. ferruginea* | 0.8 | 1 | 15 | 10 | 4 |
|  |  |  | 2 | 15 | 10 | 4 |
|  |  |  | 3 | 15 | 10 | 5 |
| 24 | *M. ferruginea* | 1 | 1 | 15 | 10 | 7 |
|  |  |  | 2 | 15 | 10 | 6 |
|  |  |  | 3 | 15 | 10 | 3 |
| 25 | *N. tabacum* | 0.4 | 1 | 1 | 10 | 0 |
|  |  |  | 2 | 1 | 10 | 0 |
|  |  |  | 3 | 1 | 10 | 0 |
| 26 | *N. tabacum* | 0.6 | 1 | 1 | 10 | 0 |
|  |  |  | 2 | 1 | 10 | 0 |
|  |  |  | 3 | 1 | 10 | 0 |
| 27 | *N. tabacum* | 0.8 | 1 | 1 | 10 | 0 |
|  |  |  | 2 | 1 | 10 | 0 |
|  |  |  | 3 | 1 | 10 | 0 |
| 28 | *N. tabacum* | 1 | 1 | 1 | 10 | 0 |
|  |  |  | 2 | 1 | 10 | 0 |
|  |  |  | 3 | 1 | 10 | 0 |
| 29 | *N. tabacum* | 0.4 | 1 | 2 | 10 | 2 |
|  |  |  | 2 | 2 | 10 | 0 |
|  |  |  | 3 | 2 | 10 | 1 |
| 30 | *N. tabacum* | 0.6 | 1 | 2 | 10 | 1 |
|  |  |  | 2 | 2 | 10 | 1 |
|  |  |  | 3 | 2 | 10 | 1 |
| 31 | *N. tabacum* | 0.8 | 1 | 2 | 10 | 2 |
|  |  |  | 2 | 2 | 10 | 0 |
|  |  |  | 3 | 2 | 10 | 2 |
| 32 | *N. tabacum* | 1 | 1 | 2 | 10 | 3 |
|  |  |  | 2 | 2 | 10 | 1 |
|  |  |  | 3 | 2 | 10 | 1 |
| 33 | *N. tabacum* | 0.4 | 1 | 3 | 10 | 3 |
|  |  |  | 2 | 3 | 10 | 0 |
|  |  |  | 3 | 3 | 10 | 0 |
| 34 | *N. tabacum* | 0.6 | 1 | 3 | 10 | 2 |
|  |  |  | 2 | 3 | 10 | 1 |
|  |  |  | 3 | 3 | 10 | 2 |
| 35 | *N. tabacum* | 0.8 | 1 | 3 | 10 | 1 |
|  |  |  | 2 | 3 | 10 | 1 |
|  |  |  | 3 | 3 | 10 | 3 |
| 36 | *N. tabacum* | 1 | 1 | 3 | 10 | 1 |
|  |  |  | 2 | 3 | 10 | 2 |
|  |  |  | 3 | 3 | 10 | 3 |
| 37 | *N. tabacum* | 0.4 | 1 | 5 | 10 | 1 |
|  |  |  | 2 | 5 | 10 | 3 |
|  |  |  | 3 | 5 | 10 | 2 |
| 38 | *N. tabacum* | 0.6 | 1 | 5 | 10 | 2 |
|  |  |  | 2 | 5 | 10 | 2 |
|  |  |  | 3 | 5 | 10 | 3 |
| 39 | *N. tabacum* | 0.8 | 1 | 5 | 10 | 3 |
|  |  |  | 2 | 5 | 10 | 2 |
|  |  |  | 3 | 5 | 10 | 4 |
| 40 | *N. tabacum* | 1 | 1 | 5 | 10 | 2 |
|  |  |  | 2 | 5 | 10 | 4 |
|  |  |  | 3 | 5 | 10 | 5 |
| 41 | *N. tabacum* | 0.4 | 1 | 10 | 10 | 3 |
|  |  |  | 2 | 10 | 10 | 4 |
|  |  |  | 3 | 10 | 10 | 2 |
| 42 | *N. tabacum* | 0.6 | 1 | 10 | 10 | 3 |
|  |  |  | 2 | 10 | 10 | 4 |
|  |  |  | 3 | 10 | 10 | 3 |
| 43 | *N. tabacum* | 0.8 | 1 | 10 | 10 | 6 |
|  |  |  | 2 | 10 | 10 | 4 |
|  |  |  | 3 | 10 | 10 | 4 |
| 44 | *N. tabacum* | 1 | 1 | 10 | 10 | 7 |
|  |  |  | 2 | 10 | 10 | 6 |
|  |  |  | 3 | 10 | 10 | 4 |
| 45 | *N. tabacum* | 0.4 | 1 | 15 | 10 | 5 |
|  |  |  | 2 | 15 | 10 | 3 |
|  |  |  | 3 | 15 | 10 | 4 |
| 46 | *N. tabacum* | 0.6 | 1 | 15 | 10 | 5 |
|  |  |  | 2 | 15 | 10 | 3 |
|  |  |  | 3 | 15 | 10 | 5 |
| 47 | *N. tabacum* | 0.8 | 1 | 15 | 10 | 3 |
|  |  |  | 2 | 15 | 10 | 7 |
|  |  |  | 3 | 15 | 10 | 6 |
| 48 | *N. tabacum* | 1 | 1 | 15 | 10 | 9 |
|  |  |  | 2 | 15 | 10 | 7 |
|  |  |  | 3 | 15 | 10 | 9 |
| 49 | *P. dodecandra* | 0.4 | 1 | 1 | 10 | 0 |
|  |  |  | 2 | 1 | 10 | 0 |
|  |  |  | 3 | 1 | 10 | 0 |
| 50 | *P. dodecandra* | 0.6 | 1 | 1 | 10 | 0 |
|  |  |  | 2 | 1 | 10 | 0 |
|  |  |  | 3 | 1 | 10 | 0 |
| 51 | *P. dodecandra* | 0.8 | 1 | 1 | 10 | 0 |
|  |  |  | 2 | 1 | 10 | 0 |
|  |  |  | 3 | 1 | 10 | 0 |
| 52 | *P. dodecandra* | 1 | 1 | 1 | 10 | 1 |
|  |  |  | 2 | 1 | 10 | 2 |
|  |  |  | 3 | 1 | 10 | 0 |
| 53 | *P. dodecandra* | 0.4 | 1 | 2 | 10 | 1 |
|  |  |  | 2 | 2 | 10 | 2 |
|  |  |  | 3 | 2 | 10 | 1 |
| 54 | *P. dodecandra* | 0.6 | 1 | 2 | 10 | 2 |
|  |  |  | 2 | 2 | 10 | 1 |
|  |  |  | 3 | 2 | 10 | 2 |
| 55 | *P. dodecandra* | 0.8 | 1 | 2 | 10 | 2 |
|  |  |  | 2 | 2 | 10 | 1 |
|  |  |  | 3 | 2 | 10 | 2 |
| 56 | *P. dodecandra* | 1 | 1 | 2 | 10 | 2 |
|  |  |  | 2 | 2 | 10 | 1 |
|  |  |  | 3 | 2 | 10 | 3 |
| 57 | *P. dodecandra* | 0.4 | 1 | 3 | 10 | 1 |
|  |  |  | 2 | 3 | 10 | 2 |
|  |  |  | 3 | 3 | 10 | 3 |
| 58 | *P. dodecandra* | 0.6 | 1 | 3 | 10 | 2 |
|  |  |  | 2 | 3 | 10 | 3 |
|  |  |  | 3 | 3 | 10 | 3 |
| 59 | *P. dodecandra* | 0.8 | 1 | 3 | 10 | 4 |
|  |  |  | 2 | 3 | 10 | 2 |
|  |  |  | 3 | 3 | 10 | 2 |
| 60 | *P. dodecandra* | 1 | 1 | 3 | 10 | 1 |
|  |  |  | 2 | 3 | 10 | 4 |
|  |  |  | 3 | 3 | 10 | 3 |
| 61 | *P. dodecandra* | 0.4 | 1 | 5 | 10 | 4 |
|  |  |  | 2 | 5 | 10 | 2 |
|  |  |  | 3 | 5 | 10 | 4 |
| 62 | *P. dodecandra* | 0.6 | 1 | 5 | 10 | 5 |
|  |  |  | 2 | 5 | 10 | 3 |
|  |  |  | 3 | 5 | 10 | 3 |
| 63 | *P. dodecandra* | 0.8 | 1 | 5 | 10 | 2 |
|  |  |  | 2 | 5 | 10 | 4 |
|  |  |  | 3 | 5 | 10 | 5 |
| 64 | *P. dodecandra* | 1 | 1 | 5 | 10 | 6 |
|  |  |  | 2 | 5 | 10 | 3 |
|  |  |  | 3 | 5 | 10 | 4 |
| 65 | *P. dodecandra* | 0.4 | 1 | 10 | 10 | 3 |
|  |  |  | 2 | 10 | 10 | 6 |
|  |  |  | 3 | 10 | 10 | 5 |
| 66 | *P. dodecandra* | 0.6 | 1 | 10 | 10 | 4 |
|  |  |  | 2 | 10 | 10 | 4 |
|  |  |  | 3 | 10 | 10 | 6 |
| 67 | *P. dodecandra* | 0.8 | 1 | 10 | 10 | 5 |
|  |  |  | 2 | 10 | 10 | 6 |
|  |  |  | 3 | 10 | 10 | 6 |
| 68 | *P. dodecandra* | 1 | 1 | 10 | 10 | 6 |
|  |  |  | 2 | 10 | 10 | 8 |
|  |  |  | 3 | 10 | 10 | 7 |
| 69 | *P. dodecandra* | 0.4 | 1 | 15 | 10 | 4 |
|  |  |  | 2 | 15 | 10 | 5 |
|  |  |  | 3 | 15 | 10 | 3 |
| 70 | *P. dodecandra* | 0.6 | 1 | 15 | 10 | 6 |
|  |  |  | 2 | 15 | 10 | 6 |
|  |  |  | 3 | 15 | 10 | 5 |
| 71 | *P. dodecandra* | 0.8 | 1 | 15 | 10 | 8 |
|  |  |  | 2 | 15 | 10 | 6 |
|  |  |  | 3 | 15 | 10 | 9 |
| 72 | *P. dodecandra* | 1 | 1 | 15 | 10 | 10 |
|  |  |  | 2 | 15 | 10 | 9 |
|  |  |  | 3 | 15 | 10 | 10 |
